# 1125. Case Study. Implementing Dynamic Axilla Splints in a Large Burn Patient

**DOI:** 10.1093/jbcr/irag033.547

**Published:** 2026-04-06

**Authors:** Amber Shojaie, Tyler Salvador, Atharva Paralikar

**Affiliations:** Children’s National Hospital, Washington, DC; Children’s National Hospital, Washington, DC; Children’s National Hospital, Washington, DC

## Abstract

**Patient Presentation (age range, injury details, relevant history):**

Burn injuries to the trunk and axilla often lead to complications such as contractures, functional limitations, and pain. Early implementation of positioning is critical for mitigating these complications and promoting functional outcomes.

**Clinical Challenges:**

Traditional static axilla splints, typically used for the burn population, limit functional use of the upper extremities and can often lead to discomfort. Dynamic axilla splints give this patient population the opportunity to progress range of motion while facilitating functional independence with opportunities for movement.

**Management Approach:**

Bilateral dynamic axilla splints were developed and implemented through a collaborative process between an occupational therapist and engineers, integrating rehabilitative principles and innovative use of 3D printed and off-the-shelf materials. A feature of the splints includes shoulder abduction adjustability to promote prolonged passive stretch through the affected axillae and cutaneous functional units. The splints incorporate a removable forearm component to enable functional use of the distal upper extremity for participation in meaningful activities, such as play and activities of daily living.

**Outcomes:**

Throughout the rehabilitation process, in both the inpatient and outpatient settings, the axilla splints have shown effectiveness in improving shoulder ROM and reducing risk for contracture. Caregiver feedback highlights the splints’ usability, comfort, and adaptability, with clinicians noting the splints’ practicality. The combination of dynamic axilla splint use with night time anti-contracture positioning aligns with rehabilitation protocols for promoting functional outcomes in the burn injury population.

**Lessons Learned:**

This case highlights the value of interdisciplinary innovation in burn rehabilitation. Dynamic axilla splints can be integrated into clinical practice to optimize contracture prevention and functional outcomes, with potential to improve both patient and caregiver experiences. Continued research is needed to validate and enhance promising initial results.

**Applicability to Practice:**

Dynamic axilla splints provide a feasible and effective alternative to static positioning devices in the burn population. Early clinical use suggests benefits for shoulder mobility, comfort, and functional independence, while supporting established rehabilitation protocols.

